# Oral Administration of Heat-Killed *Mycobacterium manresensis* Delays Progression toward Active Tuberculosis in C3HeB/FeJ Mice

**DOI:** 10.3389/fmicb.2015.01482

**Published:** 2016-01-05

**Authors:** Paula Cardona, Elena Marzo-Escartín, Gustavo Tapia, Jorge Díaz, Vanessa García, Ismael Varela, Cristina Vilaplana, Pere-Joan Cardona

**Affiliations:** ^1^Unitat de Tuberculosi Experimental, Fundació Institut Germans Trias i Pujol, CIBER Enfermedades Respiratorias, Universitat Autònoma de BarcelonaBadalona, Spain; ^2^Pathology Department, Universitat Autònoma de Barcelona, Hospital Germans Trias I PujolBadalona, Spain

**Keywords:** tuberculosis, *Mycobacterium manresensis*, tolerance, Tregs, neutrophils, C3HeB/FeJ, mice

## Abstract

Low-dose tolerance using heat-killed mycobacteria has been tested as a means of stopping progression toward active tuberculosis (TB) lesions in a human-like murine model using C3HeB/FeJ mice. In the present study, we studied the effect of different treatment schedules with heat-killed non-tuberculous-mycobacteria (NTM) species when given orally, based on the hypothesis of generating oral tolerance. This study included *M. manresensis*, a new species belonging to the *fortuitum* group, present in drinking water. Oral treatment with *M. manresensis* for 2 weeks was able to induce a PPD-specific Tregs population, which has been related to a decrease in the neutrophilic infiltration found in TB lesions. Further mechanistic analysis using PPD-stimulated splenocytes links this 2-week treatment with heat-killed *M. manresensis* to IL-10 production and memory PPD-specific Tregs, and also to a weak PPD-specific global immune response stimulation, increasing IL-6, TNF, and IFN-γ production. In lungs, this treatment decreased the bacillary load, granulomatous infiltration and pro-inflammatory cytokines (TNF, IFN-γ, IL-6, and IL-17). Oral administration of *M. manresensis* during standard treatment for TB also significantly reduced the relapse of active TB after ending the treatment. Overall the data suggest that the use of heat-killed *M. manresensis* could be a new and promising tool for avoiding active TB induction and as adjunctive to TB treatment. This supports the usefulness of generating a new kind of protection based on a complex balanced immune response focused on both destroying the bacilli and including control of an excessive inflammatory response.

## Introduction

Tuberculosis (TB) is a major global health problem, with 9 million new TB cases and 1.5 million deaths reported in 2013 (WHO, 2014). It is estimated that one third of the world's population is infected with latent tuberculosis infection (LTBI). Fortunately, only a small proportion of infected subjects go on to develop the disease. Although, some host factors, mainly immunosuppression conditions such as human immunodeficiency virus (HIV) coinfection, drug abuse, renal failure or diabetes mellitus, are known to be related to generation of the disease, it still remains unclear why almost half of healthy adult cases develop TB.

As reviewed recently (Vilaplana and Cardona, 2014), research efforts to develop new treatment or preventive strategies have focused on destruction of the bacilli by identifying metabolic pathway targets (development of antimicrobial agents) or studying how the host's immune system identifies it (new vaccine development), whereas little effort has been invested in trying to modulate the host's response against the bacilli, which is paramount for understanding the development of infectious diseases (Casadevall and Pirofski, 2003), with TB not being an exception (Cardona, 2010).

Understanding the infection's progression to active TB is essential for finding new strategies to fight TB. In this regard, induction of liquefaction in TB lesions has been identified as a key factor that allows extracellular growth of the bacilli and the development of cavities, thereby favoring spread of the infection (Grosset, 2003; Cardona, 2011). For this reason, we developed a murine model of active TB using a C3HeB/FeJ mice strain in which lesions liquefact, thus mimicking human-like progression (Marzo et al., 2014). Characterization of this model showed us that extracellular bacillary growth starts before the liquefaction process and is fuelled by the constant attraction of neutrophils and the induction of Neutrophilic Extracellular Traps (NETs), thereby starting a local and uncontrollable bacillary spread. This, in turn, allows fast growth of the granulomas, the induction of new lesions and the coalescence of neighboring lesions, which are responsible for the logarithmic growth of the granulomas. Once a certain size is exceeded, the center of the granulomas first develops caseous necrosis and then the caseum softens, thus leading to liquefaction (Marzo et al., 2014; Vilaplana and Cardona, 2014). In this regard, a recent review of the pathology of human lesions based on necropsies from the pre-antibiotic era also highlights the key role of neutrophils as inducers of exudative lesions, which are clearly responsible for the progression toward active TB (Cardona, 2015). In summary, we can conclude that the onset of active TB is a consequence of an excessive inflammatory response and the coalescence of different lesions (Vilaplana and Cardona, 2014).

Evidence for the key role of inflammation in progression to TB disease has also been reported by others. Thus, a detrimental role of excessive accumulation of neutrophils has been described in mice (Eruslanov et al., 2005; Keller et al., 2006) and, in humans, genetic studies performed with TB and leprosy patients indicated that insufficient but also excessive inflammation are key to TB pathogenesis (Tobin et al., 2010). Similarly, Berry et al. found a signature of active TB that measures interferon IFN type I-inducible genes in whole blood (Berry et al., 2010). Unexpectedly, the genes induced in susceptible population were expressed in neutrophils and not in T-cells, thus suggesting that over-activation of neutrophils by IFNs may contribute to TB pathogenesis.

Host-Directed Therapies (HDT) are currently becoming increasingly popular, as reviewed by Hawn et al. (2013), Zumla et al. (2015), and Zumla and Maeurer (2015). In this regard, we previously tested ibuprofen (Vilaplana et al., 2013) and other non-steroidal anti-inflammatory drugs (NSAID) (Marzo et al., 2014) in the C3HeB/FeJ model, with very promising results. We are now exploring their potential therapeutic use in humans, but as NSAIDs can produce adverse effects related to dose and length of treatment, these should be taken into account when considering long-term therapy (Michels et al., 2012; Bjarnason, 2013).

After the success obtained with the aforementioned anti-inflammatory treatment, and for safety reasons, we decided to look for a strategy that avoids the potential toxicity of these drugs and the perspective of constant administration to obtain an effective prophylactic effect. The prophylactic/therapeutic use of low dose tolerance to avoid an excessive inflammatory response has been widely used by different authors in the field of experimental autoimmune encephalitis (Ochi et al., 2006), diabetes mellitus and other autoimmune diseases (Weiner et al., 2011), as well as infectious diseases (Levy and Ilan, 2007) and atherosclerosis (Harats et al., 2002). The role of regulatory T cells (Tregs) in TB has been controversial as they were initially thought to fuel progression toward active TB. However, recent reports seem not to support this idea (Green et al., 2010; Leepiyasakulchai et al., 2012), thus leading to a more neutral role in their interference against the Th1 response.

## Methodology

### Experimental design

We first conducted a proof-of-concept study to assess the role of Tregs in protection against the development of active TB by comparing the susceptible strain (C3HeB/FeJ) with the resistant one (C3H/HeN). We then tested the usefulness of low-dose heat-killed *Mycobacterium tuberculosis* (Mtb) cells and their protective role. Finally, we tested different environmental NTM species in order to ensure any possible transfer of this strategy to the market and further characterized the effect of the species that provided the best results.

### Animals

Female C3HeB/FeJ and C3H/HeN specific-pathogen-free mice (6–8 weeks old) were obtained from Jackson Laboratories (Bar Harbor, Maine, USA) and Harlan Labs (Castellar del Vallès, Catalonia, Spain). All procedures were conducted in a BL3 security facility. Mice were infected with 2 × 10^4^ CFU of *M. tuberculosis* H37Rv Pasteur strain via the caudal vein.

All procedures were performed according to protocol DMAH6119, which was reviewed by the Animal Experimentation Ethics Committee of the Hospital Universitari Germans Trias i Pujol (registered as B9900005) and approved by the Dept d'Agricultura, Ramaderia, Pesca, Alimentació i Medi Natural of the Catalan Government, according to current national and European Union legislation regarding the protection of experimental animals (Law 1997 of the Catalan Government; Spanish Royal Decree 1201/2005; and European legislation 86/609/EEC; 91/628/EEC; 92/65/EEC and 90/425/EEC).

Mice were supervised daily and euthanized, if required, with isoflurane (inhalation excess), following a strict protocol, in order to ensure animal welfare.

### Study of the role of Tregs in protection against the development of active TB

To study the role of Tregs in TB, three experiments were performed with C3HeB/FeJ and C3H/HeN mice. First, 6 mice from each strain were infected and sacrificed at 3 weeks post-infection to evaluate Treg population in spleen. This experiment was repeated with a final time point of week 2 post-infection, the splenocytes collected being cultured for 7 days. In the third experiment, a total of 12 C3H/HeN mice were infected, and one group was Treg-depleted *in vivo* by administration of anti-mouse CD25 antibody (Clone PC61.5, eBioscience Inc. San Diego, CA) the day before the infection. Blood was collected at different time points to evaluate Treg depletion by flow cytometry. Animals were sacrificed at day 46 post-infection to evaluate lung histopathology.

### Treatment preparation

Different mycobacteria from the Experimental Tuberculosis Unit strain collection, namely *M. tuberculosis, M. kansasii, M. avium*, and *M. manresensis* (new species, Rech et al., 2015 CECT 8638) were used to prepare the treatments. *M. bovis* BCG Danish (Pfizer Inc., NY, USA) was also used. Bacteria were grown in 7H11 plates (BCG, *M. kansasii*, and *M. manresensis*) or in Proskauer-Beck broth (*M. tuberculosis* and *M. avium*) and subcultured in Proskauer-Beck broth (or in 7H11 plates, for *M. manresensis)* in aeration and agitation at 37°C. The bacillary load (BL) of each culture was determined by serial dilution and culture on 7H11 plates, and a Blood Agar plate was also seeded to rule out contamination. Cultures were then inactivated by heating at 80°C for 60 min. Sterilization was confirmed by negative culture in 7H11 (10 plates), Blood Agar, McConkey Agar, and Saboureaud Agar. The inactivated cultures were diluted 1:1 in sterile sucrose (10% sucrose in water) and aliquoted in 1 ml vials for storage at −80°C.

### Assessment of treatments

The effect of treatment on the survival of infected mice was studied. This assessment was done for each treatment when given prophylactically and therapeutically at different doses (10^3^–10^6^ heat-killed bacilli/animal) and administration schedules (every day, every other day, three times-a-week). The results presented here were repeated 3 times, using 10 C3HeB/FeJ mice per group (a total of 120 animals).

In order to further characterize the effect of treatment, we evaluated several parameters at week 3 post-infection. For this purpose a total of 20 C3HeB/FeJ mice were used (5 per group of treatment). Animals received seven oral doses of *M. tuberculosis* (10^5^ heat-killed bacilli/animal), BCG (10^6^ heat-killed bacilli/animal), and *M. manresensis* (10^5^ heat-killed bacilli/animal) every other day from the day of infection and were sacrificed at day 21 post-infection. At the final time point, pathology and BL in lungs, and effect on T cell populations were studied (see Sections 2.6 to 2.8). A control group was included and treated with the corresponding dilution of the excipient (mannitol).

The oral administration of heat-killed mycobacteria as a coadjuvant therapy to the human standard treatment against active TB (a combination of rifampicin, isoniazid, ethambutol, and pyrazinamide (RHEZ)) was also studied. A total of 21 C3HeB/FeJ mice were used. They started to be treated at week 4 post-infection, when their weight started to decrease. All animals received a commercial combination of the four drugs (RIMSTAR®, Sandoz Farmaceutica, Barcelona) adjusted to their weight, for a total of 4 weeks. Half of the animals also received heat-killed *M. manresensis* (10^5^ heat-killed bacilli/animal) orally, 5 days a week for a total of 6 weeks (in addition to the 4-week-treatment with RHEZ, plus 2 more weeks). At the final time point, pathology in lungs was studied (see Section Lung Pathology).

### Bacillary load

Samples of lung lobes from each animal were collected, homogenized and several dilutions plated on nutrient Middlebrook 7H11 agar (BD Diagnostics, Spark, USA). The number of CFU was counted after incubation for 28 days at 37°C and the results expressed as CFU/mL.

### Lung pathology

Lungs were fixed in 10% buffered formalin, embedded in paraffin and 5-μm sections stained with haematoxylin-eosin (HE), Masson trichromic (MTC) or Ziehl-Neelsen (ZN) stain for microscopic observation and histometric analysis using the NIS-Elements D version 3.0x software package (Nikon Instruments Inc., Tokyo, Japan). Eight recuts of half-lung of each mice stained with HE were used to determine the damaged area as a percentage of total lung area.

### Immuno-characterization

#### Cell isolation and cell cultures

Spleens were mechanically disrupted and filtered through a 40-μm cell strainer (BD Diagnostics, Spark, USA), with erythrocytes being incubated for 8 min in lysis buffer (Tris 17 mM, NH_4_Cl 0.14 M). 10^6^ cells were either directly stained for flow cytometric analysis or cultured, depending on the experiment. Cell culture was conducted in supplemented RPMI 1640 (10% Fetal Calf Serum, streptomycin 100 μg/ml, penicillin 100 U/ml, 2-mercaptoethanol 0.025 mM, sodium pyruvate 1 mM) in 24-well plates at 37°C and 5% CO_2_, with or without PPD stimuli (final concentration of 10 μg/ml; Statens Serum Institute, Kobenhavn, Denmark). After 24 h, 4 days, or 7 days of culture, the content of each well was harvested and stained for flow cytometric analysis. Culture supernatants were stored at −80°C for further study of the cytokine profile. Lungs were snap-frozen when collected and kept stored at −80°C until processed for cytokine profile analysis. They were then subjected to mechanical disruption and homogenized with lysis buffer (sodium azide 0.05%, Triton X-100 0.5%, protease inhibitor cocktail from Sigma at 1:500, in PBS; Sigma-Aldrich Co. LLC, St. Louis, MO, USA).

#### PPD-specific T cell population analysis by flow cytometry

Mouse Regulatory T Cell Staining Kit #2 (eBioscience Inc., SD, USA) was used for intracellular staining (Foxp3) according to the manufacturer's indications. For membrane staining, cells were incubated for 30 min at 4°C with the antibodies, followed by fixation with 4% formaldehyde in PBS for 10 min at room temperature.

PPD-specific T cell populations, obtained from spleen cultures at 24 h, 4 days, or 7 days, were studied by flow cytometry. Different CD4+ T cell types, namely CD25+CD39+ Regulatory Memory T cells; CD25-CD39+ Effector Memory T cells, ex-Tregs IL-17 producers; CD25+CD39− T Effector cells and CD25-CD39− Naïve T cells, according to Dwyer et al.'s characterization (Dwyer et al., 2010), were studied.

The antibodies used were anti-mouse CD4 FITC, anti-mouse CD25 PE and anti-mouse Foxp3 APC (eBioscience Inc., SD, USA), anti-mouse CD3e BV™421, anti-mouse CD25-PerCP-Cy^TM^5.5 (BD biosciences, CA, USA) and anti-mouse CD39 PE (BioLegend Inc., CA, USA).

**Figure 1:**
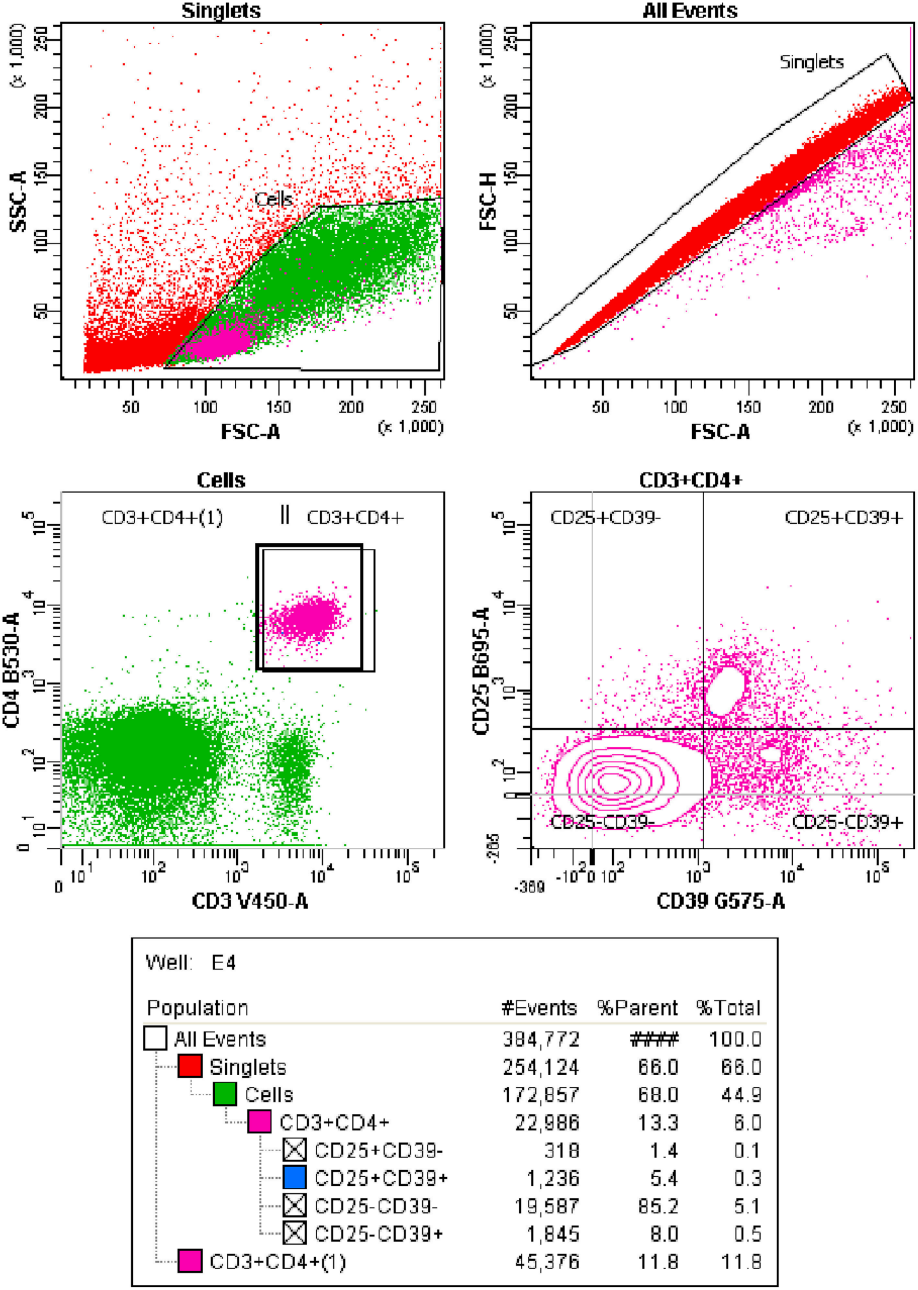
**Flow cytometry analysis carried out**. CD3+CD4+ T cells were classified into 4 groups: CD25+CD39+ Regulatory Memory T cells; CD25-CD39+ Effector Memory T cells, ex-Tregs IL-17 producers; CD25+CD39− T Effector cells and CD25-CD39− Naïve T cells.

Once stained, samples were read in a flow cytometer (BD LSRFortessa™, BD Biosciences, CA, USA). Data were analyzed using FACSdiva™ software (BD Biosciences, CA, USA; Figure 1).

#### Cytokine profile analysis

A cytokine profile study was performed in splenocyte culture supernatant and lung homogenates. The following cytokines were measured by Luminex xMAP® technology: IFNγ, TNFα, IL-5, IL-6, IL-10, IL-13, and IL-17. Results were expressed as pg per ml of supernatant. The assay was performed with the MILLIPLEX® MAP kit (EMD Millipore Corporation, Billerica, MA, USA) following the manufacturer's instructions and analyzed with xPONENT Software (Luminex Corporation, Austin, TX, USA).

### Data analysis

GraphPad Prism version 5.00 for Windows, (GraphPad Software, San Diego California USA) was used for graphics and statistics, with differences of *p* < 0.05 being considered to be statistically significant.

## Results

### Protective role of regulatory T cells (Treg)

**Figure 2:**
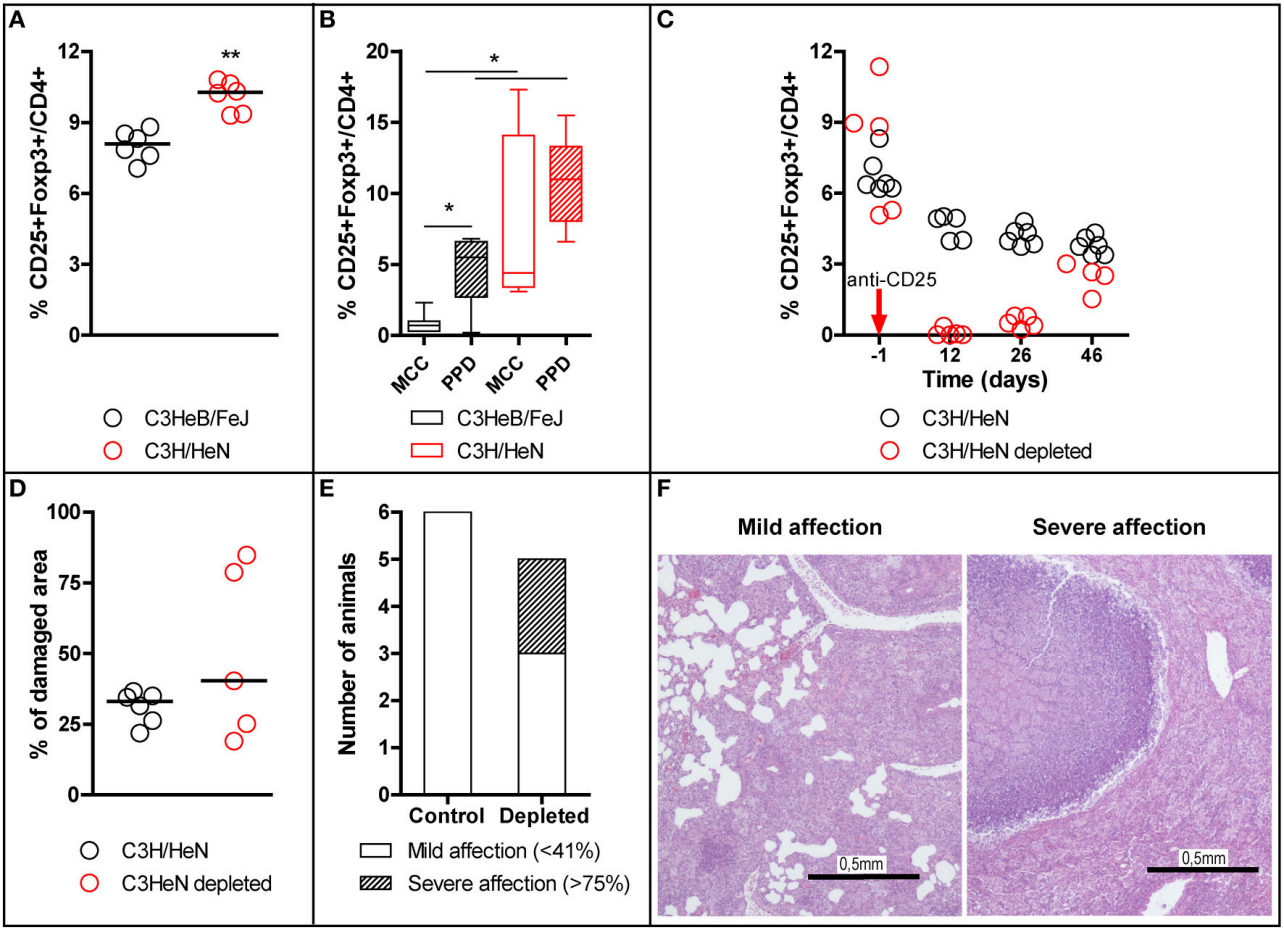
**Role of Treg in TB-resistant C3H/HeN mice strain**. Treg Foxp3+ population was studied in C3HeB/FeJ and C3H/HeN mice in splenocytes **(A,B)**. Splenocytes obtained on day 21 post-infection **(A)**, statistically significant differences (^**^p = 0.0022, Mann Whitney test); each circle represents an animal and the median is represented with a line. Splenocytes obtained on day 14 post-infection were cultured for 7 days with PPD stimulus (PPD) or with no stimulation (MCC) **(B)**, statistically significant differences between groups (^*^p < 0.05; Mann Whitney test); the boxplot shows the median, quartiles and minimum and maximum values. A Treg-depletion study was performed and lung pathology was studied in depleted and undepleted animals **(C–F)**. Treg depletion was confirmed by measuring the percentage of Treg Foxp3+ cells at different time points **(C)**. Percentage of damaged area out of total lung area **(D)**; each circle represents an animal and the lines are medians. Proportion of animals with mild or severe affection in lungs in control undepleted group and Treg depleted group **(E)**; bars indicate number of animals. H/E stained lung recuts showing mild and severe infection **(F)**.

In order to study the mechanisms that modulate the inflammation in active TB, the Treg population was studied in C3HeB/FeJ mice and compared with the TB-resistant mouse strain C3H/HeN. Mice from both strains were infected IV and the Treg population in spleen studied by flow cytometry 3 weeks post-infection. As shown in Figure 2A, the median percentage of Treg (CD4+CD25+Foxp3+ cells) out of total CD4+ in spleen was higher in C3H/HeN than in C3HeB/FeJ mice (C3H/HeN: 10.28%, C3HeB/FeJ: 8.09%; *t*-test, *p* = 0.0022). The same experiment was repeated with the animals being sacrificed at day 14 and the resulting splenocytes cultured and PPD-stimulated. The C3H/HeN resistant mice had higher basal and PPD-specific Treg percentage in culture compared to C3HeB/FeJ mice, as shown in Figure 2B. Intergroup differences were statistically significant (*p* < 0.05, Mann Whitney test).

In the third experiment, C3H/HeN mice were depleted of Treg cells by administration of anti-CD25 prior to Mtb infection. All animals treated were effectively depleted of Treg cells, as shown in Figure 2C, except for one animal, which was excluded from the analysis. One animal from the Treg-depleted group was sacrificed at day 32 for ethical reasons as it exhibited a marked TB infection in the lungs. The remaining animals were sacrificed at day 46 as scheduled and their lung pathology assessed by qualitative and quantitative (histometry) histological analysis. The histometry showed differences in the percentage of damaged lung area between both groups (not statistically significant) (Figure 2D). The results for the Treg-depleted group were highly scattered: three animals had a similar percentage of damaged lung area to the controls (20–40%), whereas the other two animals had a higher percentage of damaged area (around 80%). The qualitative analysis matched these findings: the animals with mild TB infection (less than 40% of damaged lung area) presented medium size lesions with no signs of necrosis or other forms of cell death, with mainly macrophage and lymphocyte infiltration, and scattered neutrophils around foamy macrophages, while those with severe TB infection (more than 75% of damaged lung area) presented very big lesions with central caseous necrosis and liquefaction (Figure 2F). When classifying the animals according to the degree of lung affection it was found that the frequency of severe affection (>75% of damaged lung) was 0 in the control group and 2/5 in the depleted group (Figure 2E; *p* < 0.1, *z*-test of proportions, one-tailed).

Taken together, these results suggest a protective role of regulatory T cells in our model.

### Oral administration of inactivated mycobacteria spp. increased mice survival

**Figure 3:**
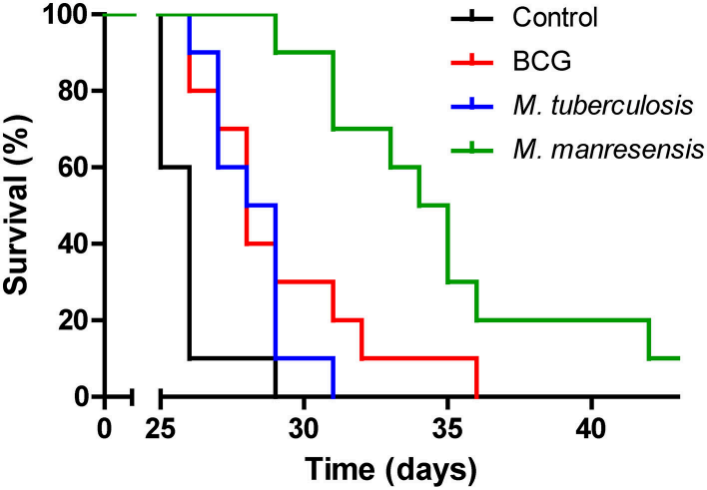
**Effect of treatment on survival**. The survival curves for three different oral treatments are represented together with the survival curve for the untreated control group. Infected C3HeB/FeJ mice were treated every other day for 2 weeks from infection day with 10^5^–10^6^ heat-killed bacilli/animal. Statistically significant differences were observed between untreated mice and mice treated with heat-killed BCG (*p* = 0.0038), Mtb (*p* = 0.0029), or *M. manresensis* (*p* < 0.0001) (Log-rank Test).

*Mycobacterium tuberculosis* (Mtb) heat-killed bacilli were administered orally to C3HeB/FeJ infected mice to test their influence on disease progression. Treatment was found to increase survival of the mice in a statistically significant manner. Different doses and administration patterns were tested and positive results were obtained both when administered pre-infection or post-infection (data not shown). The survival curve of a representative experiment is shown in Figure 3.

Other mycobacterial species, including *M. bovis* Bacille-Calmette-Guérin (BCG) and environmental species, were also tested. Animals treated with heat-killed BCG, *M. kansasii* (data not shown) and *M. manresensis*, a newly discovered environmental mycobacteria species belonging to the *M. fortuitum* complex (CECT 8638) (Rech et al., 2015) showed an increased survival compared to untreated controls (Figure 3). This protection was also achieved when giving the same treatment with the same concentration of living *M. manresensis* (data not shown). As was the case for Mtb, different doses and administration schedules were tested for all of these strains and positive results were obtained both when administered pre-infection or post-infection (data not shown). Oral treatment with *M. avium* was also tested but found not to improve mice survival (data not shown).

### Oral administration of heat-killed mycobacteria reduced lung pathology

**Figure 4:**
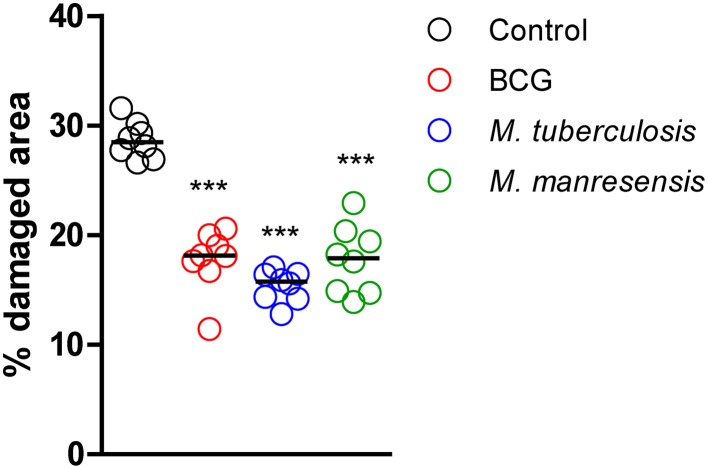
**Effect of treatment on lung pathology: histometric analysis**. Infected C3HeB/FeJ mice were treated every other day for 2 weeks from infection day with 10^5^–10^6^ heat-killed bacilli/animal. The percentage of damaged area out of total lung area was studied in each treatment group on samples obtained on day 21 post-infection. Each circle represents an animal and the lines are medians. Statistically, significant differences were observed between untreated mice and mice treated with heat-killed mycobacteria (^***^p = 0.0002; Mann Whitney test).

**Figure 5:**
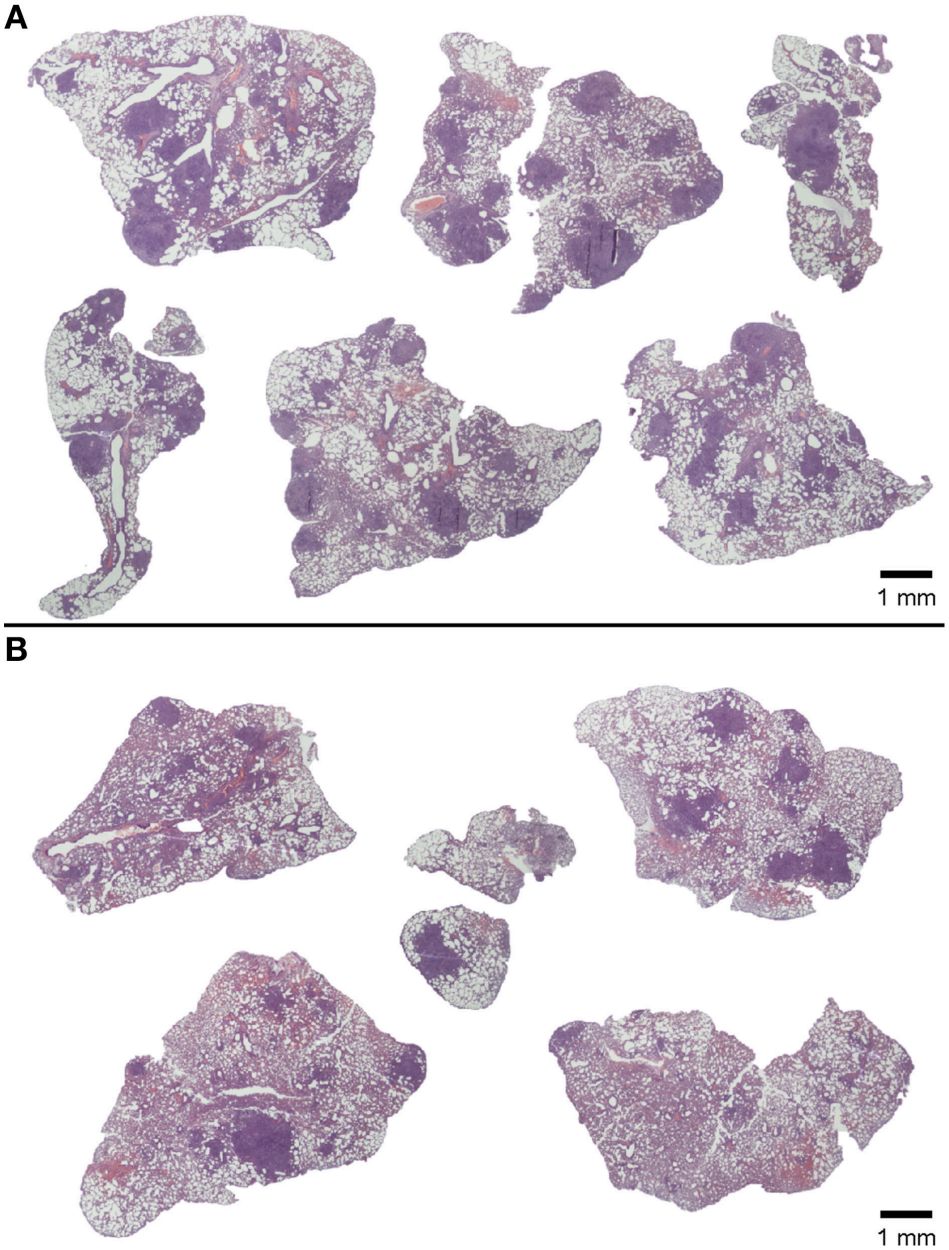
**Effect of treatment on lung pathology: macroscopic view of lesions**. HE-stained lung recuts of untreated control mice **(A)** or mice treated with heat-killed *M. manresensis* **(B)** of samples obtained on day 21 post-infection.

The effect on lung pathology was evaluated for oral treatments with three different heat-killed mycobacteria, namely Mtb, BCG and *M. manresensis*. Figure 4 shows how all treatments significantly reduced the infiltration in the parenchyma (statistically significant differences: *p* = 0.0002; Mann Whitney test). Figure 5 shows one of the H/E recuts, which was used to measure the percentage damaged area, for the untreated control group and the group treated with *M. manresensis*. In agreement with the histometry analysis, the picture enclosed in Figure 5 shows the presence of fewer and smaller lesions in the lungs of treated mice than in the untreated control group.

### Oral administration of heat-killed mycobacteria decreases lung bacillary load

**Figure 6:**
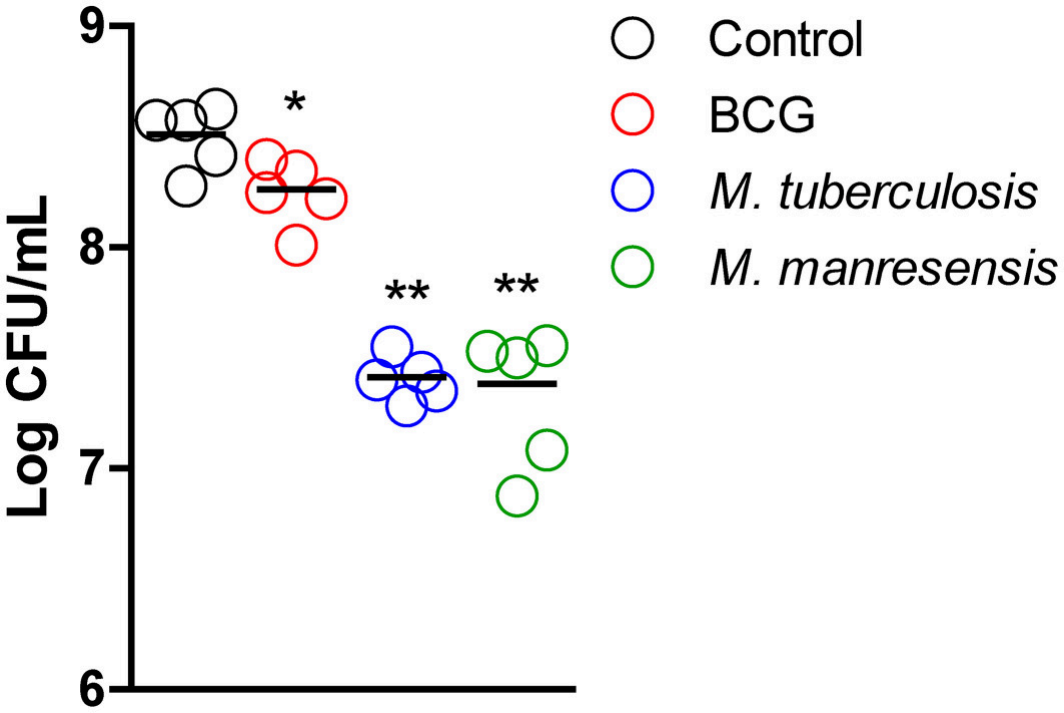
**Effect of treatment on bacillary load in lungs**. Infected C3HeB/FeJ mice were treated every other day for 2 weeks from infection day with 10^5^–10^6^ heat-killed bacilli/animal. BL in lungs obtained on day 21 post infection is expressed as log CFU/ml. The boxplot shows the median, quartiles and minimum and maximum values, with a different pattern for each treatment group. Statistically, significant differences were observed between treated and untreated control mice (BCG: ^*^p = 0.0317, Mtb: ^**^p = 0.0079, *M. manresensis*: ^**^p < 0.0079; Mann Whitney test).

The effect on bacillary load in lungs was evaluated for oral treatments with three different heat-killed mycobacteria, namely Mtb, BCG and *M. manresensis*. The three treatments decreased the BL in lungs at day 21 post-infection in a statistically significant manner (BCG *p* = 0.0317, Mtb *p* = 0.0079, *M. manresensis p* = 0.0079; Mann Whitney test) as shown in Figure 6.

### Immuno-characterization of heat-killed mycobacteria treatment

**Figure 7:**
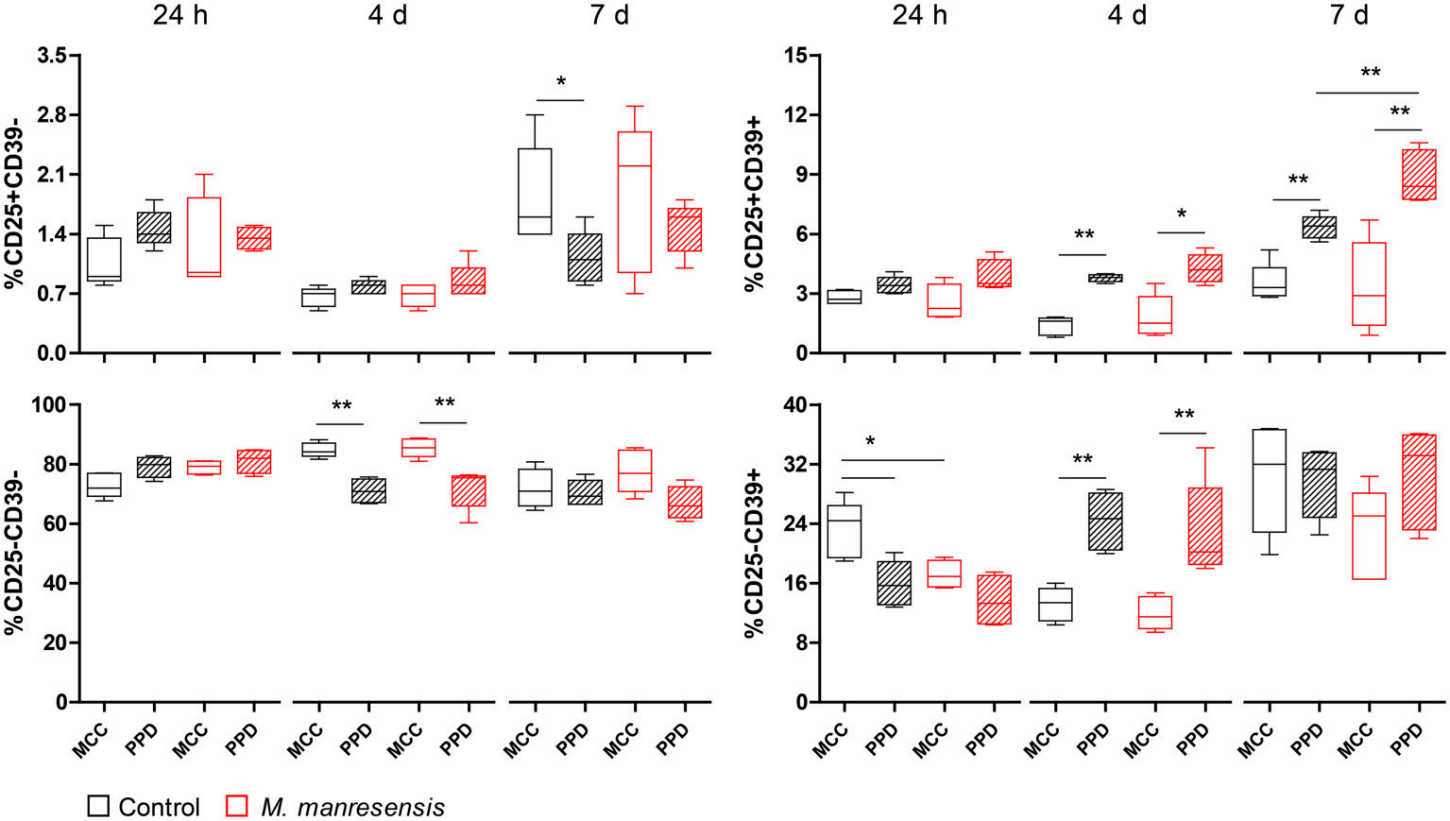
**Effect of treatment with heat-killed *M. manresensis* on T cell populations in the spleen**. Percentage of the four populations defined by CD25 and CD39 markers (out of the total of CD3+CD4+ cells). Splenocytes *obtained on day 21 post-infection were* cultured for 24 h, 4 days, or 7 days with PPD stimulus (PPD) or without stimulation (MCC). The boxplot shows the median, quartiles and minimum and maximum values, with a different color for each treatment. Statistically, significant differences are marked with asterisks (^*^p < 0.05, ^**^p < 0.01; Mann Whitney test).

The immunomodulation achieved by the oral treatment with heat-killed Mtb, BCG and *M. manresensis* was also characterized. Flow cytometric analysis of splenocyte cultures was used to assess different CD4+ T cell populations (M&M, Section PPD-specific T cell population analysis by flow cytometry). The results are shown in Figure 7 (*M. manresensis* treatment) and Supplementary Image 1 (Mtb, BCG, and *M. manresensis* treatments). The naïve CD25-CD39− population, which represents the higher percentage of CD3+CD4+ cells, tended to be higher under unstimulated conditions than under PPD stimulation. In contrast, the CD25+CD39− population, which represented approximately 2% of CD3+CD4+ cells, did not show any major differences between stimulated and non-stimulated culture conditions. A mild increase in this population was observed when cells were cultured for 7 days.

We also observed this trend in CD25+CD39+ Regulatory T cells, which represent around 2–9% of all CD3+CD4+ cells. In contrast to naïve T cells, this population tends to be higher under PPD stimulation. Furthermore, a higher PPD-specific response of CD25+CD39+ was observed in splenocytes from mice treated with *M. tuberculosis* and *M. manresensis* in comparison to untreated mice, but only when cells were cultured for 7 days (statistically significant differences: Mtb *p* = 0.0159, *M. manresensis p* = 0.0079; Mann Whitney test).

The CD25-CD39+ population accounts for about 20% of all CD4+ T cells. A higher percentage of PPD-stimulated cells (with statistically significant differences) is observed when cultured for 4 days, with levels of both stimulated and unstimulated cells decreasing after culture for 7 days.

### Cytokine profile

**Figure 8:**
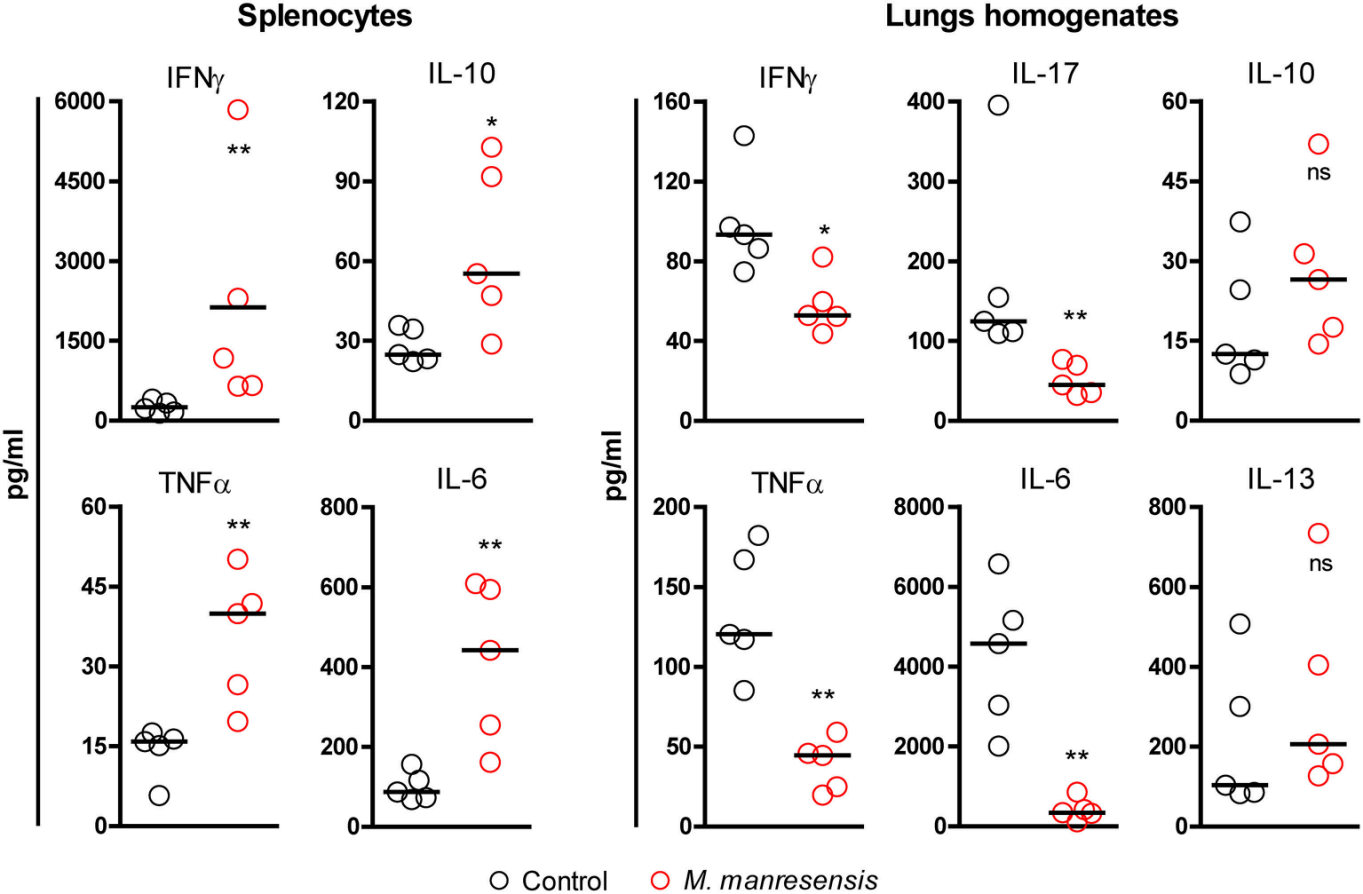
**Cytokine profile in spleen and lungs**. Effect of treatment of infected C3HeB/FeJ mice every other day for 2 weeks with heat-killed *M. manresensis*. Samples were obtained on day 21 post-infection. Each circle represents an animal and the median is represented with a line. Cytokine levels are expressed as pg/ml. Statistically, significant differences are marked with asterisks (^*^p < 0.05, ^**^p < 0.01; Mann Whitney test).

We studied the presence of different cytokines in the splenocyte supernatant and lung homogenates of control untreated mice or mice treated with heat-killed *M. manresensis*. Mice treated with *M. manresensis* showed higher levels of pro-inflammatory cytokines but a reduced inflammatory milieu in lungs when compared to control mice. Detailed results are shown in Figure 8. In splenocyte culture, treated mice showed a statistically significant increase in IFNγ, TNFα, IL-6, and IL-10 levels in spleens, whereas they showed a statistically significant decrease in IFNγ, TNFα, IL-6, and IL-17 levels in lung homogenates.

IL-5 levels could not be detected in any sample, thus suggesting a poor Th2 response.

### Heat-killed mycobacteria as coadjuvant therapy

**Figure 9:**
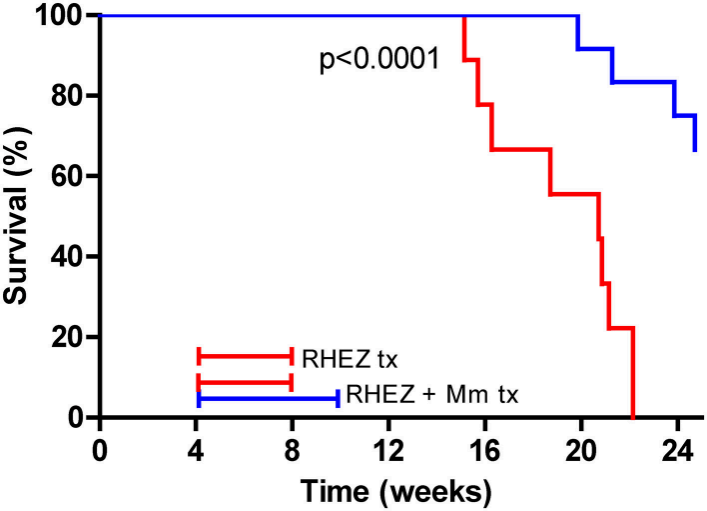
**Heat-killed *M. manresensis* as a coadjuvant therapy**. Survival curves for infected C3HeB/FeJ mice treated with RHEZ (red line) or RHEZ in combination with heat-killed *M. manresensis* (Mn) (blue line). Treatment started at week 4 post-infection, RHEZ therapy was administered for 4 weeks and Mm therapy lasted 6 weeks.

**Figure 10:**
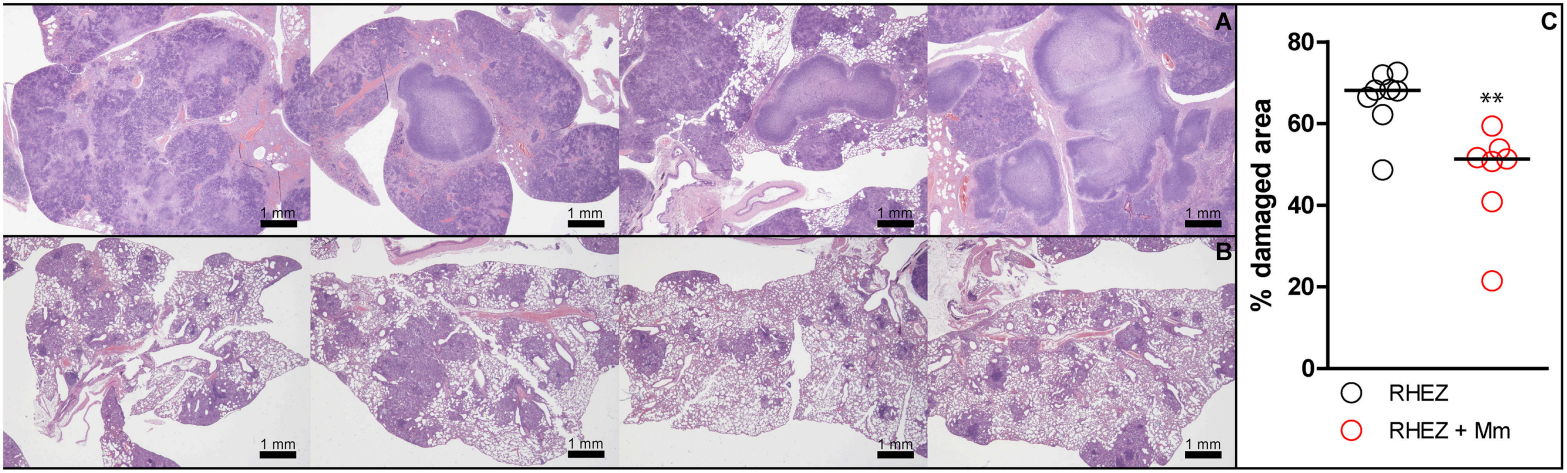
**Heat-killed *M. manresensis* as a coadjuvant therapy: histometric analysis**. Macroscopic view of HE-stained lung recuts from mice treated with RHEZ **(A)** or treated with RHEZ and heat-killed *M. manresensis* **(B)**. The percentage of damaged area out of total lung area was studied in both groups **(C)**. Each circle represents an animal and the lines are medians. Statistically, significant differences were observed between untreated mice and mice treated with heat-killed mycobacteria (^**^p = 0.059; Mann Whitney test).

**Figure 11:**
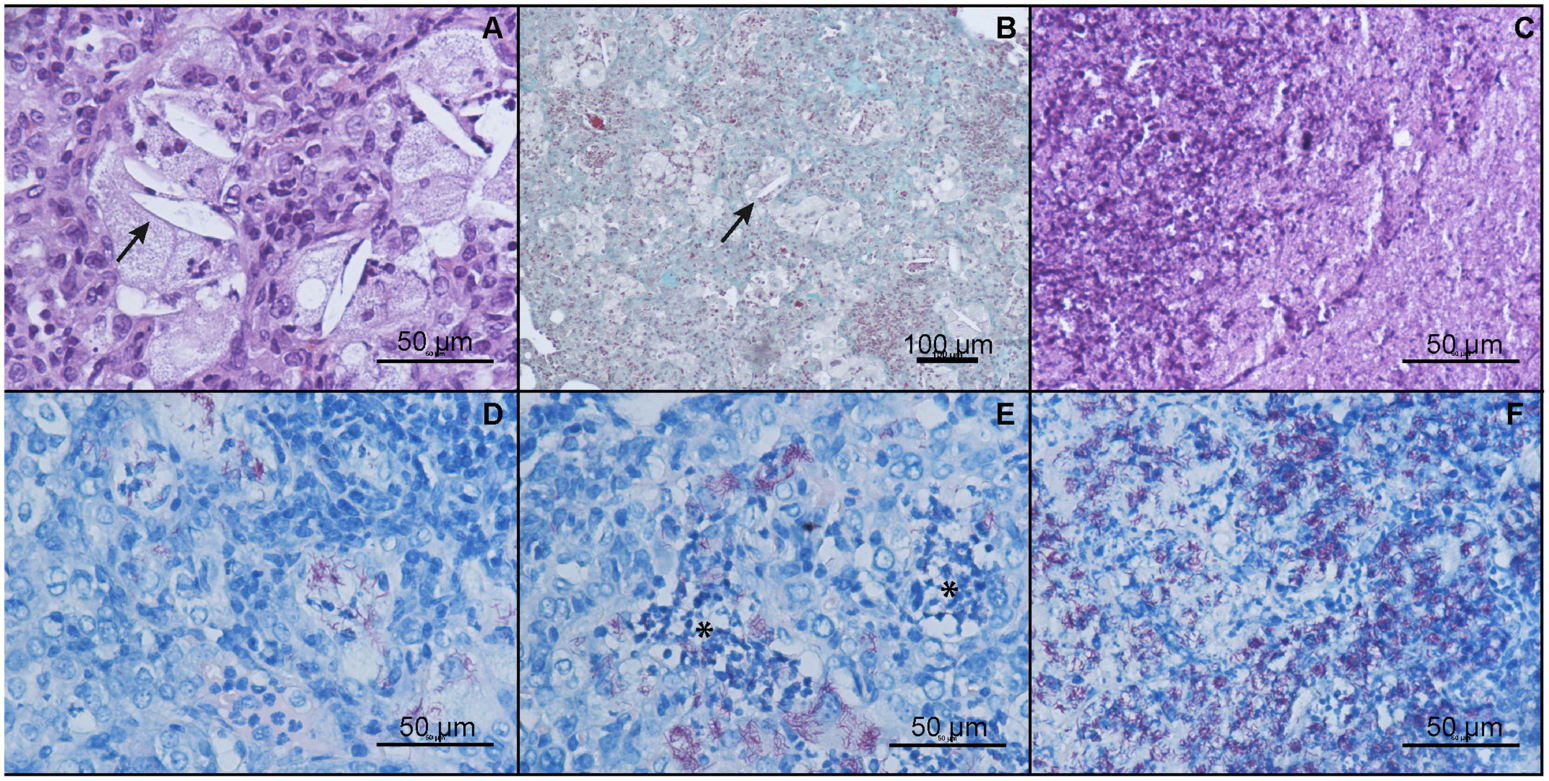
**Heat-killed *M. manresensis* as a coadjuvant therapy: qualitative histological analysis**. Pulmonary infiltration in mice treated with RHEZ and heat-killed *M. manresensis* at week 24 post-infection **(A,B,D,E)** is compared with mice only treated with RHEZ that had to be euthanized according to the welfare monitoring control **(C,F)**. Pictures **(A)** and **(B)** show the haematoxylin-eosin (HE) and Masson trichromic (MTC) stains respectively showing the structure of murine chronic TB infection with a lot of foamy macrophages and where cholesterol crystals (marked with arrows) can be seen. MTC stain showing the honeycomb patron caused by the progressive fibrosis originated at the alveolar wall. Pictures **(D)** and **(E)** show the localization of bacilli with the Ziehl-Neelsen (ZN) stain, initially only in foamy macrophages **(D)** to growth on PMN rafts (marked with asterisks) afterwards **(E)**. Picture **C** shows the accumulation of cellular debris, which is the main property of the infiltration in terminal mice, after HE stain. Massive bacillary presence is shown with ZN stain in picture **(F)**.

The effect of heat-killed *M. manresensis* on mice survival when administered as coadjuvant treatment to RHEZ was evaluated, with *M. manresensis*-treated mice showing a statistically significant increase in survival (*p* < 0.0001, Log-rank test; Figure 9). Comparison of the histopathology between the animals from the control group that had to be euthanized according to the welfare monitoring control (between weeks 15 and 22 post-infection) with the survivors from the *M. manresensis*-treated group that were euthanized to terminate the experiment (week 24) showed a clear difference in terms of intrapulmonary infiltration (51 vs. 68%; *p* = 0.0059, Mann Whitney test; Figure 10). Looking at the quality of the lesions (Figure 11), all the samples of the control group showed a massive necrosis with liquefaction in the center and big patches of massive accumulation of nuclear debris plenty of bacilli. In the case of the group treated with *M manresensis* lesions resembled granulomas of chronic tuberculosis infection seen in resistant mice (C57BL/6) where the bacilli are accumulated in foamy macrophages (FM), and where big cholesterol crystals can be seen as described before (Cardona et al., 2003; Cáceres et al., 2009). There is also a progressive fibrosis of the parenchyma caused by the proliferation of the fibroblasts of the alveolar wall, in a honeycomb pattern as described by Dunn and North (1996). The difference is that there are little infiltrations of PMN, especially around the infected FM. Different animals have lesions with different degrees of evolution, and it can be seen how this PMN infiltration become bigger and is infected with bacilli, that multiplies over them, as described before (Marzo et al., 2014).

## Discussion

The data provided herein suggest, for the first time, that a low dose tolerance regimen (i.e., administration of 10^5^ heat-killed *M. manresensis* daily for 14 days) could be used to control progression toward active TB. This treatment regimen seems to interfere with the excessive inflammatory response, a fact that appears to be key to the development of human-like lesions (Cardona, 2015), and thus is also able to reduce the bacillary concentration in lungs (Marzo et al., 2014). In our opinion, this could be related to the induction of Tregs.

It is well established that dose and route of administration determine the immune response to an administered antigen (Faria and Weiner, 2005), therefore we have good reason to think that our regimen induces tolerance to the mycobacteria bacilli. Continuous oral administration modifies the response triggered by the infection through Tregs, as has also been seen in other infectious or autoimmune diseases (Harats et al., 2002; Ochi et al., 2006; Levy and Ilan, 2007; Weiner et al., 2011).

We have shown that treatment with heat-killed bacilli is effective for reducing both the infiltration area and the bacillary load in lungs, in addition to increasing survival. As such, we then decided to further characterize the effect of treatment on the immune system to prove that tolerance is induced.

In this regard we first investigated the role of Tregs in our model as it was feasible that oral tolerance could be articulated through this type of cell. The role of Tregs in Mtb infection is controversial, with some authors considering the induction of Tregs to be detrimental (Ribeiro-Rodrigues et al., 2006; Chiacchio et al., 2009) but others pointing to a protective role for Tregs (Leepiyasakulchai et al., 2012). To investigate the role of Tregs in our experimental system we compared the Treg population in C3HeB/FeJ mice with their positive controls (C3H/HeN mice), which share the MHC haplotype but have a different susceptibility to Mtb (Marzo et al., 2014). We found that the better outcome was associated with a stronger regulatory immune response in the C3H/HeN strain, as the Treg percentage was higher (also PPD-specific response), and when we depleted C3H/HeN mice of Treg by administration of anti-CD25 the susceptibility to Mtb increased. Two out of five mice developed necrotic lesions, whereas in several experiments conducted by our group with this mouse strain, no animals have either died or developed such necrotic lesions. Although, T CD4+CD25+ cells include Tregs as well as potential T CD4+CD25+CD39− Th subsets (Dwyer et al., 2010), the latter account for about 1% of all T CD4+, therefore Treg depletion is far more relevant. Taken together, these results strongly support the hypothesis that Tregs are protective in TB, therefore we continued our research by studying the effect of treatment with heat-killed mycobacteria on Tregs.

As we were interested in the induction of memory Tregs, we designed the concept of 7 days incubation, together with a study of the CD39 marker. We believe this marker to be very relevant as it has been related to both the memory phenotype and induction of tolerance (Borsellino et al., 2007; Dwyer et al., 2010; Roberts et al., 2014). Furthermore, unlike Foxp3, it is a surface marker, which translates into less aggressive cell processing and easier and faster protocols. Other relevant T cell subtypes, such as T memory effector cells CD25-CD39+, which have shown to play a detrimental role in organ transplantation in humans, or T effector cells, both of which are capable of secreting IL-17, and thus having a pro-inflammatory profile (Dwyer et al., 2010), were also studied with the marker combination used. Apart from being used in the study of autoimmune diseases, this CD25 CD39 marker combination has been validated as a Treg marker in the study of TB, exhibiting a negative correlation with IL-17 T cells in peripheral blood (Chiacchio et al., 2009; de Cassan et al., 2010).

Use of these markers showed an increase in Treg cells in splenocyte cultures from treated mice, together with a slight global stimulation. The levels of IFN-γ, TNF, and IL-6 were increased in cultured spleen samples from mice treated with *M. manresensis*. This suggest a global stimulation that also includes an increase in the immunosuppressive cytokine IL-10, which can be related to the increase in Treg, as seen by other authors who used 10^6^ heat-killed *M. chelonae* intraperitoneally once a week for 3 weeks (Ho et al., 2010). In that study, the authors also demonstrated a parallel increase in IFN-γ, although they did not check for IL-6 or TNF. The most important aspect, however, is that the increase in IL-6 was not linked to the presence of IL-17 (Gao et al., 2009), which was undetectable, and this is logical as an exaggerated inflammatory response with neutrophilic attraction is not seen in the spleen in the C3HeB/FeJ model, where there is also effective control of bacillary load (data not shown). This could be due to the fact of the presence of Treg counterbalancing IFN type I production (Srivastava et al., 2014; Aida et al., 2014), thus favoring the increased IFNγ response (Manca et al., 2001); or because of the lack of PMN infiltration in the spleen prevents the production of type I IFN (Berry et al., 2010), thus favoring the presence of both Tregs and IFN-gamma in this organ in the context of Mtb infection. However, further experiments would be needed to investigate this issue.

A lower inflammatory milieu was found in the lungs of mice treated with *M. manresensis* when compared to control animals, with lower levels of IFNγ, TNFα, IL-17, and IL-6. In our opinion, the population of PPD-specific memory Tregs is attracted to the lungs and must be crucial for reducing the inflammatory response in situ, specially by counterbalancing the Th17 response (Zhou et al., 2008; Zheng et al., 2013).

Lung histopathology of mice treated with RHEZ shows how the addition of heat-killed *M. manresensis* is able to stop the progression of the lesions, favoring the fibrosis of the tissue which might contribute to create an anti-inflammatory milieu able to abrogate the growth of the bacilli by curtailing the infiltration of the lesion with monocytes or PMN. This phenomenon is amplified in big-mammals thanks to the stimulation of the fibroblasts of the intralobar septae, that encapsulate the lesions at very early stages, promoting this anti-inflammatory milieu and stopping the bacillary growth (Gil et al., 2010; Cardona, 2015).

The modulatory effect of NTM was demonstrated years ago after intravenous inoculation was shown to induce a non-specific cellular immune response (Collins, 1971) and protect against subsequent aerosol Mtb infection to a similar degree as in BCG vaccinated mice (Orme and Collins, 1984). On the other hand, subcutaneous sensitization with *M. avium* interfered with BCG vaccination by stopping its growth, although this was not seen after sensitization with *M. chelonae* or *M. fortuitum* (Brandt et al., 2002). The recent study by Poyntz et al. (2014) is interesting as it shows how important the route of administration for NTM is (in this case, *M. avium*). An increase in Th1 response can be detected when inoculating heat-killed IP several times, thereby increasing the efficacy of BCG, whereas oral administration of living bacilli tended to increase Th2 response in the lung during Mtb infection and reduced the protection against Mtb infection. Treatment with heat-killed *Mycobacterium vaccae*, which has been used for the treatment of active TB (von Reyn et al., 2010) and has recently been reviewed by Gröschel et al. (2014), deserves particular attention due to its ability to reduce Th2 responses and increase Th1 ones.

Finally, it is relevant to recall that studies in BCG-vaccinated infants in the United Kingdom or in Malawi showed no evidence that the initial response to NTM affected the vaccine-induced change in IFN-γ response (Weir et al., 2006). In this regard, it is interesting to note that, to date, no surrogates of protection have been found in TB. Th1 responses, including poly-functional cells, have received attention for a number of years, although an evaluation of their protective value in a large BCG trial in South Africa showed that protected and unprotected newborns exhibited an equivalent immunological profile (Kagina et al., 2010). In this sense, our paper may also help to shed some light on other biomarkers that are more closely related to the induction of a balanced immune response and are able to avoid an excessive inflammatory response than can lead to active TB, instead of focussing on an immune response that exclusively targets destruction of the bacilli.

In conclusion, induction of a balanced immune response triggered by PPD-specific memory Tregs through the administration of heat-killed *M. manresensis* has demonstrated an ability to protect against the progression of Mtb infection to active disease and relapse after TB treatment. This has been demonstrated in the experimental model induced in C3HeB/FeJ mice, which reproduce “human-like” lesions. This finding might help to focus on a new kind of “host-directed” prophylactic and therapeutic approach as well as the development of new predictive biomarkers.

## Author contributions

EM and PJC had substantially contributed to the conception and design of the work. PC, EM, GT, JD, VG, IV, CV, and PJC contributed to the acquisition, analysis, and interpretation of data for the work. All authors contributed to drafting the work and revising the work; and gave final approval of the version to be published. All authors agreed to be accountable for all aspects of the work in ensuring that questions related to the accuracy or integrity of any part of the work are appropriately investigated and resolved.

## Funding

This project was funded by the Plan Nacional I+D+I co-financed by ISCIII-Subdirección General de Evaluación and Fondo-EU de Desarrollo Regional (FEDER) and cofinanced through the Projects PI11/01702 and PI14/01038 and MS 13/00174, and the contracts CP13/00174 and IFI14/00015.

### Conflict of interest statement

“Inactivated mycobacteria for oral use in the prevention of tuberculosis” is a patent (PCT/ES2013/000145) owned by IGTP and CIBER Enfermedades Respiratorias and invented by PC, CV, and EM. Manremyc sl is a new spin-off from IGTP and CIBER Enfermedades Respiratorias that has been created exclusively for the development of this patent. PC is the CEO of this spin-off. The reviewer JT and handling Editor declared their shared affiliation, and the handling Editor states that the process nevertheless met the standards of a fair and objective review.

## Supplementary material

Supplementary Image 1 **Effect of treatment with different heat-killed mycobacteria treatments on T cell populations in the spleen**. Percentage of the four populations defined by CD25 and CD39 markers (out of the total of CD3+CD4+ cells). Splenocytes obtained on day 21 post-infection were cultured for 24 h, 4 days, or 7 days with PPD stimulus (PPD) or without stimulation (MCC). The boxplot shows the median, quartiles and minimum and maximum values, with a different color for each treatment. Statistically significant differences are marked with asterisks (^*^*p* < 0.05, ^**^*p* < 0.01; Mann Whitney test).
