# Supplementary figures and images for: Oral Administration of Heat-Killed Mycobacterium manresensis Delays Progression toward Active Tuberculosis in C3HeB/FeJ Mice
Source: Front Microbiol. 2016 Jan 5;6:1482. doi: 10.3389/fmicb.2015.01482 (PMC4700139; doi:10.3389/fmicb.2015.01482)

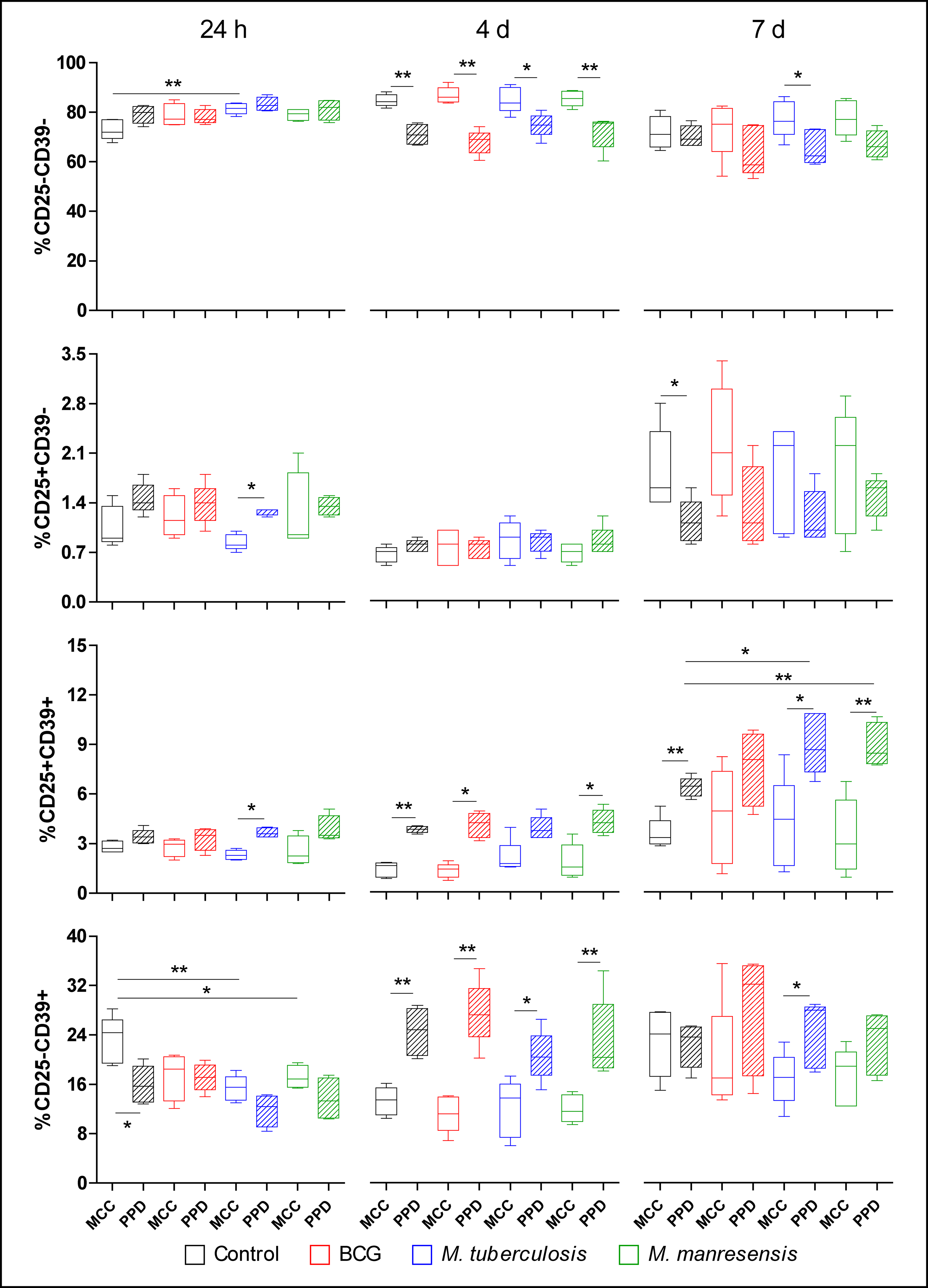

Supplement: Supplementary Image 1 — Effect of treatment with different heat-killed mycobacteria treatments on T cell populations in the spleen. Percentage of the four populations defined by CD25 and CD39 markers (out of the total of CD3+CD4+ cells). Splenocytes obtained on day 21 post-infection were cultured for 24 h, 4 days, or 7 days with PPD stimulus (PPD) or without stimulation (MCC). The boxplot shows the median, quartiles and minimum and maximum values, with a different color for each treatment. Statistically significant differences are marked with asterisks (*p < 0.05, **p < 0.01; Mann Whitney test). [file Image1.tif]
